# Dissecção Espontânea da Artéria Coronária: Existem Diferenças entre Homens e Mulheres?

**DOI:** 10.36660/abc.20210550

**Published:** 2022-12-20

**Authors:** Teresa Alvarado, Marcos García-Guimaraes, Juan Manuel Nogales, Marcelo Jimenez-Kockar, Fernando Macaya, Fernando Alfonso

**Affiliations:** 1 Hospital Universitário de La Princesa Madri Espanha Hospital Universitário de La Princesa, Madri – Espanha; 2 Hospital Universitário del Mar Barcelona Espanha Hospital Universitário del Mar, Barcelona – Espanha; 3 Hospital Universitário de Badajoz Badajoz Espanha Hospital Universitário de Badajoz, Badajoz – Espanha; 4 Hospital Universitário de la Santa Creu i Sant Pau Barcelona Espanha Hospital Universitário de la Santa Creu i Sant Pau, Barcelona – Espanha; 5 Hospital Universitário Clínico San Carlos Madri Espanha Hospital Universitário Clínico San Carlos, Madri – Espanha

**Keywords:** Dissecção das Artérias, Síndrome Coronariana Aguda, Diversidade de Gênero, Displasia Fibromuscular, estudo Multicêntrico

## Introdução

A dissecção espontânea da artéria coronária (DEAC) é uma causa pouco comum, mas cada vez mais reconhecida da síndrome coronariana aguda (SCA), que afeta principalmente as mulheres. É a principal causa da SCA em relação à gravidez e em até um terço das mulheres com >50 anos de idade.^1 , 2^ Devido ao perfil demográfico único e à baixa prevalência de fatores de risco cardiovascular tradicionais, sua causa parece ser multifatorial, com contribuições de fatores genéticos, influências hormonais, arteriopatias herdadas ou adquiridas e doenças inflamatórias sistêmicas.^1^ A DEAC é definida como uma separação espontânea (não iatrogênica) das camadas da parede arterial coronariana. Dois importantes mecanismos poderiam explicar a fisiopatologia da DEAC. No primeiro, o evento primário é uma hemorragia na mídia sem ruptura intimal; no segundo, o evento primário é a formação de um rompimento que leva a um retalho intimal. Essa ruptura inicial leva à formação de um hematoma intramural ou um lúmen verdadeiro e falso que pode causar isquemia miocárdica.^1 , 2^ Nossa compreensão dessa doença melhorou muito na última década, como resultado dos esforços internacionais de pesquisa.^1 , 2^ Entretanto, como aproximadamente 90% dos casos afetam as mulheres, as características da DEAC nos homens e as possíveis diferenças clínicas entre os sexos continuam pouco estabelecidas.^3^ Segundo dados anteriores, homens com DEAC têm fatores de predisposição e precipitação diferentes das de mulheres. A presença de displasia fibromuscular (DFM) e a associação com distúrbios mentais parecem ser menos comuns em homens, enquanto que o exercício físico intenso é mais comumente observado como desencadeador da DEAC nesse grupo.^4 , 5^ O objetivo desse estudo foi comparar características básicas, apresentação clínica, características angiográficas, estratégias de manejo e curso hospitalar entre homens e mulheres com DEAC.

## Métodos

O Registro espanhol da DEAC (SR-SCAD) (NCT03607981) é um estudo multicêntrico prospectivo de âmbito nacional sobre DEAC realizado sob os auspícios da Associação de Cardiologia Intervencionista da Sociedade Espanhola de Cardiologia. O protocolo específico, assim como o formulário de relato de caso e o consentimento informado, foram aprovados pelo Comitê de Ética do centro coordenador (Hospital Universitário La Princesa, Madri), de acordo com a legislação espanhola vigente. De junho de 2015 a abril de 2019, 344 pacientes consecutivos com DEAC (387 lesões) foram incluídos de 31 centros espanhóis. Todos os angiogramas coronarianos foram cuidadosamente revisados em conjunto por 2 profissionais especializados no laboratório central do centro coordenador, usando uma metodologia pré-definida para análise da DEAC.^6^ Após cuidadosa revisão dos angiogramas e dos dados clínicos, 26 pacientes foram excluídos devido a um diagnóstico alternativo mais provável. O tipo de DEAC foi caracterizado utilizando a classificação angiográfica Saw.^7^ As lesões com aparência de duplo lúmen foram classificadas como Tipo 1. O Tipo 2 foi definido como uma mudança abrupta no calibre arterial com demarcação clara do diâmetro normal para o estreitamento difuso. As lesões com estreitamento focal, parecidas com lesões ateroscleróticas, foram classificadas como Tipo 3. Para a definição de outros padrões angiográficos, tais como a morfologia “inseto bastão” ou “rabanete”, seguiu a descrição inicial de Motreff et al.^8^ A presença de outros achados angiográficos sugestivos de DEAC, tais como um padrão de “linha quebrada” também foi sistematicamente avaliado. Finalmente, a análise da tortuosidade coronária foi realizada de acordo com a definição da Clínica Mayo.^9^

As variáveis contínuas são expressas como média ± desvio padrão, e as variáveis categóricas como frequências e porcentagens. Na comparação, utilizaram-se os testes qui-quadrado ou exato de Fisher, como requerido para dados categóricos, e o teste *t* de Student para variáveis contínuas.

## Resultados

Comparou-se um total de 39 homens e 279 mulheres com DEAC ( Tabela 1 ). A idade foi semelhante em ambos os grupos e a maioria dos pacientes apresentava alguns fatores clássicos de risco cardiovascular. O consumo de drogas recreativas foi significativamente maior nos homens (26% vs 3%; p<0,01) enquanto o hipotireoidismo foi mais comum nas mulheres (15% vs 3%; p=0,04). O estresse emocional e físico eram os desencadeadores numericamente mais comuns nas mulheres e nos homens, respectivamente, mas sem diferenças estatisticamente significativas. A maioria dos pacientes apresentava-se sob a forma de infarto do miocárdio sem elevação do segmento ST. Os homens apresentavam mais frequentemente arritmias ventriculares como sintoma inicial (8% vs 1,4%; p= 0,01) e também durante a internação (5% vs 0,4%; p<0,01) do que as mulheres ( Tabela 1 ). Não houve diferenças na detecção de malformações vasculares extracoronarianas (MVEs) ou DFM entre os homens e mulheres que se submetem à triagem para essa patologia.

**Tabela 1:** Diferenças entre os sexos na DEAC: Características basais e apresentação clínica

	Homens (n=39)	Mulheres (n=279)	p
Idade (anos)	50±10	54±11	0,3
Alguns fatores de risco cardiovascular	32 (82%)	216 (77%)	0,5
Hipertensão arterial	12 (31%)	106 (38%)	
Dislipidemia	14 (36%)	97 (35%)	
Diabetes mellitus	2 (5%)	14 (5%)	
Drogas recreativas	10 (26%)	9 (3%)	<0,01
Hipotireoidismo	1 (3%)	41 (15%)	<0,01
Depressão	8 (20%)	57 (20%)	1
Ansiedade	4 (10%)	51 (18%)	0,2
Estresse emocional	5 (13%)	74 (26%)	0,06
Exercício físico intenso	7 (18%)	36 (13%)	0,4
IAMSSST	20 (51%)	150 (54%)	0,8
IAMCSST	12 (31%)	113 (40%)	0,3
Arritmias ventriculares			
Sintoma inicial	3 (8%)	4 (1,4%)	0,01
Internação	2 (5%)	1 (0,4%)	<0,01

*IAMSSST: infarto do miocárdio sem elevação do segmento ST; IAMCSST: infarto do miocárdio com elevação do segmento ST.*

**Figura 1:**
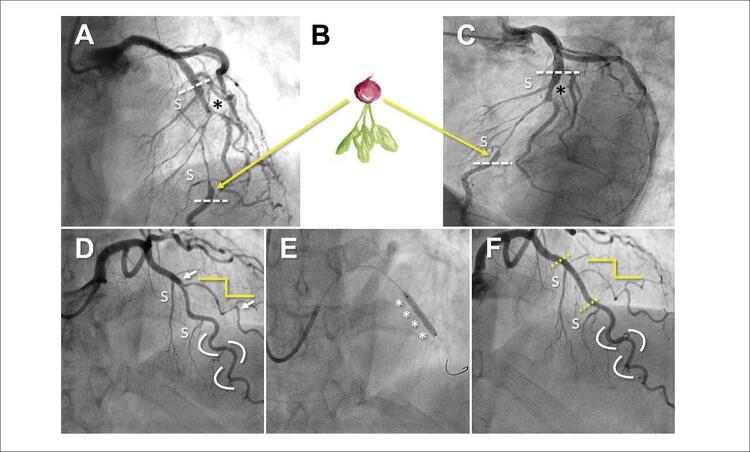
Painel superior. (A-C) Vista angiográfica cranial direita (A) e esquerda (C) em um homem de 45 anos, sem fatores de risco cardiovascular, admitido por NSTEMI. Observa-se lesão extensa na ADA esquerda leve, característica de DEAC com IMH tipo 2a (delimitada por linhas brancas tracejadas), que também afeta o óstio do segundo ramo diagonal (asterisco preto). O IMH começa e termina após a formação de dois ramos septais (S). Na porção final do IMH há evidência de uma mudança abrupta no calibre do vaso gerado pela compressão gerada pelo próprio IMH e recuperação distal do calibre normal. Essa morfologia lembra uma cauda de rabanete (morfologia “rabanete”) (B) descrita nesta entidade. Considera-se também a ausência de tortuosidade coronária significativa nesse homem com DEAC. Painel inferior. (D-F) Projeções angiográficas com angulação craniana de uma mulher de 65 anos, ex-fumante, com hipertensão e dislipidemia, admitida por IAMSSST, com dados eletrocardiográficos sugestivos de isquemia anterior. (D) Redução do calibre na ADA esquerda leve e uma extensa lesão no primeiro ramo diagonal (limitada por setas brancas) compatível com IMH tipo 2a. Destaca a presença de um segmento distal saudável da ADA esquerda com tortuosidade significativa, enquanto o ramo diagonal afetado apresenta uma acentuada “retificação” dos ângulos suaves na curvatura arterial, com um padrão de “linha quebrada” (linha amarela quebrada) também descrito nessa patologia. Decidiu-se implantar diretamente um stent farmacológico (E, asteriscos) com um bom resultado final (F, segmento delimitado por linhas amarelas), mas com uma discreta deterioração no ramo diagonal, devido à extensão do IMH. IMH: hematoma intramural; ADA: artéria coronária descendente anterior; IAMSSST: infarto do miocárdio sem elevação do segmento ST; DEAC: dissecção espontânea da artéria coronária.

Notavelmente, as características angiográficas foram significativamente diferentes entre os sexos. Os homens eram mais propensos a apresentar leves anomalias coronarianas angiográficas compatíveis com aterosclerose das coronárias associada em outros territórios (15% vs 4%; p<0,01), enquanto apresentavam menos frequentemente tortuosidade das artérias coronárias (36% vs 72%, p<0,01). Além disso, alguns padrões angiográficos característicos previamente descritos nesta entidade,^8^ tais como a morfologia “rabanete invertido” e a terminação da DEAC pouco antes da formação do ramo lateral, foram mais frequentemente observados em homens. Por outro lado, a morfologia “inseto bastão” e “linha quebrada” ocorreu principalmente em mulheres. ( Tabela 2 ) ( Figura 1 ).

**Tabela 2:** Diferenças entre os sexos na DEAC: características angiográficas

	Homens (n=39)	Mulheres (n=279)	p
IVUS/OCT	8 (20%)	25 (9%)	0,03
ADA esquerda	16 (41%)	133 (48%)	0,9
Aterosclerose coronária	6 (15%)	10 (3,6%)	<0,01
Tortuosidade coronária	14 (36%)	201 (72%)	<0,01
Tipo 1 Saw	11 (28%)	59 (21%)	0,4
Tipo 2 Saw	22 (56%)	172 (62%)	0,5
Morfologia “Rabanete”	3 (7,7%)	41 (15%)	0,2
Morfologia “Rabanete invertido”	6 (15%)	12 (4%)	<0,01
DEAC termina antes da formação do ramo lateral	4 (10%)	7 (2,5%)	0,03
Morfologia “linha quebrada”	1 (3%)	52 (19%)	0,01
Morfologia “inseto bastão”	0 (0%)	26 (9%)	0,05

*IVUS: ultrassom intravascular; ADA: artéria descendente anterior; OCT: tomografia de coerência óptica ; DEAC: dissecção espontânea da artéria coronária.*

O tratamento foi principalmente o manejo médico conservador em ambos os grupos. Entretanto, a intervenção coronariana percutânea (ICP) foi usada como tratamento primário em 22% das mulheres e 23% dos homens. Os procedimentos mais comuns de ICP foram o implante de *stent* farmacológico (61% em mulheres e 67% em homens), a angioplastia simples com balão (20% em mulheres e 22% em homens), e o implante de dispositivo biorreabsorvível (13% em mulheres e 11% em homens) ( Tabela 3 ). A taxa de sucesso da ICP foi alta em ambos os sexos (86% em mulheres e 100% em homens), sem diferenças entre eles. Tanto no manejo conservador quanto no intervencionista, não foram observadas diferenças entre os sexos na incidência do desfecho clínico combinado (incluindo morte hospitalar, reinfarto, insuficiência cardíaca e acidente vascular cerebral) ( Tabela 3 ). Independentemente da estratégia de manejo, o tratamento na alta hospitalar também foi similar em ambos os grupos, embora os betabloqueadores fossem mais comumente usados em mulheres (81% vs. 65%; p= 0,03).

**Tabela 3:** Diferenças entre os sexos na DEAC: estratégias de manejo e curso hospitalar

	Homens (n=39)	Mulheres (n=279)	p
Presença de MVEs (inclusive DFM)	2/8 (25%)	29/85 (34%)	0,2
Tratamento conservador	30 (77%)	217 (78%)	0,9
Tratamento intervencionista	9 (23%)	61 (22%)	0,8
Stent farmacológico	6 (67%)	37 (61%)	
Angioplastia com balão	2 (22%)	12 (20%)	
Dispositivo biorreabsorvível	1 (11%)	8 (13%)	
Evento hospitalar adverso*	4 (10%)	14 (5%)	0,2

MVEs: malformações vasculares extracoronarianas; DFM: displasia fibromuscular. *Inclui: morte, reinfarto, insuficiência cardíaca e acidente vascular cerebral.

## Discussão

Considerando a escassez de informações sobre a DEAC em homens, este registro nacional prospectivo fornece novos dados interessantes que complementam as evidências anteriores. As descobertas mais importantes deste estudo são as diferenças entre homens e mulheres em relação a fatores precipitantes e achados angiográficos. O consumo de drogas recreativas e arritmias ventriculares foram encontrados com mais frequência em homens. A maior prevalência de hipotireoidismo nas mulheres é provavelmente apenas um reflexo do fato de que o hipotireoidismo afeta predominantemente as mulheres. Com relação aos achados angiográficos, os homens frequentemente apresentavam irregularidades sugerindo a possibilidade de uma aterosclerose leve subjacente e significativamente menos tortuosidade coronária do que as mulheres. Havia também diferenças em certos padrões angiográficos típicos dessa entidade. A morfologia “rabanete invertido” e a terminação da DEAC pouco antes da formação de um ramo lateral eram mais comuns nos homens, enquanto a morfologia “inseto bastão” e da “linha quebrada” ocorria principalmente nas mulheres. Embora a proporção de pacientes examinados para MVEs em nosso registro fosse relativamente baixa (29% de todos os pacientes), não foram encontradas diferenças na incidência de MVEs entre homens e mulheres com DEAC. É importante ressaltar que a falta de um estudo sistemático de triagem neste sentido nos homens pode se dever à percepção de que a DFM é uma doença que também afeta principalmente as mulheres. Entretanto, Fahmy et al.,^4^ sugeriram a importância do rastreamento sistemático de MVEs em homens, semelhante ao que é atualmente recomendado para as mulheres.^2^ De acordo com a literatura contemporânea, os eventos adversos eram pouco comuns em ambos os grupos, e os pacientes apresentavam um bom resultado intra-hospitalar, a maioria deles sob manejo conservador. Entretanto, a ICP às vezes é necessária, e parece ser uma boa opção para pacientes selecionados de alto risco, sem diferenças no curso hospitalar entre homens e mulheres. No entanto, ainda são necessários estudos maiores e seguimento mais longo para avaliar possíveis diferenças de resultados entre os sexos.

Estudos anteriores comparando homens e mulheres com DEAC foram estudos retrospectivos de um único centro envolvendo um número menor de pacientes.^4 , 5 , 10^ O trabalho atual é o primeiro estudo prospectivo, nacional e multicêntrico sobre DEAC e inclui um número maior de pacientes (39 homens e 279 mulheres), com foco nas diferenças entre os sexos. Em alguns estudos comparativos entre os sexos,^4 , 5 , 10^ os homens com DEAC eram mais jovens que as mulheres. No entanto, não foram encontradas diferenças relacionadas à idade em nossa coorte. Como a DEAC não é uma doença aterosclerótica, parece razoável esperar uma idade semelhante para homens e mulheres. No entanto, ainda é possível que o diagnóstico de DEAC seja mais comumente negligenciado em homens mais velhos. Como em relatórios anteriores,^4 , 5^ o abuso de drogas foi identificado como um gatilho para a DEAC em homens. No entanto, em contraposição a estudos anteriores, em nosso estudo não foram observadas diferenças significativas nos estressores físicos e emocionais.^4 , 5^

Sharma et al.,^5^ sugeriram que os homens provavelmente apresentavam uma aparência angiográfica de duplo lúmen (71% DEAC Tipo 1 vs 21% DEAC Tipo 2). No entanto, em nosso estudo, consistentemente com dados anteriores,^1 , 2^ a DEAC Tipo 2 foi o padrão angiográfico mais prevalecente em ambos os sexos. É razoável especular que homens com padrões de DEAC diferentes da clássica “dissecção” angiográfica são mais vezes subdiagnosticados. Motreff et al.,^8^ descreveram padrões angiográficos específicos em mulheres com DEAC. Pela primeira vez, descrevemos aqui esses padrões angiográficos em relação a sexo. Embora sejam desconhecidos os mecanismos subjacentes pelos quais essas morfologias angiográficas únicas são geradas, a identificação desses padrões interessantes pode ajudar no diagnóstico diferencial e também pode sugerir diferenças fisiopatológicas potenciais entre homens e mulheres com DEAC.

Algumas limitações deste estudo observacional devem ser reconhecidas. Primeiro, embora este seja um grande estudo prospectivo e multicêntrico (o maior até hoje na Europa), a DEAC é uma condição rara, e alguns vieses de seleção podem ter ocorrido devido ao diagnóstico negligenciado e ao tamanho relativamente pequeno da amostra. Em segundo lugar, a porcentagem de pacientes examinados para MVEs foi baixa em nosso registro. Terceiro, as imagens intracoronarianas não foram obtidas rotineiramente neste registro, apesar de seu valor diagnóstico e capacidade de ajudar a entender as diferenças angiográficas entre sexos. Quarto, a DEAC é provavelmente subdiagnosticada em homens idosos e também naqueles em que a apresentação da DEAC tem um padrão angiográfico atípico. Finalmente, analisaram-se apenas os eventos durante a hospitalização. Embora este registro nacional projeta obter dados de seguimento clínico a longo prazo, essa informação não está disponível atualmente.

A identificação das diferenças entre homens e mulheres com DEAC proporciona novos elementos para nossa compreensão da patologia dessa entidade clínica única e da DEAC em geral. Deveríamos ter em mente que a DEAC é uma doença que também afeta os homens e que ambos os sexos se beneficiam de um manejo inicialmente conservador com um excelente desfecho hospitalar.
