# Supplementary material for: Dissecção Espontânea da Artéria Coronária: Existem Diferenças entre Homens e Mulheres?
Source: Arq Bras Cardiol. 2022 Dec 20;120(1):e20210550. [Article in Portuguese] doi: 10.36660/abc.20210550 (PMC9833275; doi:10.36660/abc.20210550)
Supplement: Translation

# Translation: Spontaneous Coronary Artery Dissection: Are There Differences between Men and Women?

**Authors:** Teresa Alvarado, Marcos García-Guimaraes, Juan Manuel Nogales, Marcelo Jimenez-Kockar, Fernando Macaya, Fernando Alfonso

**Affiliations:** 1 Spain Hospital Universitario de La Princesa, Madrid – Spain; 2 Spain Hospital Universitario del Mar, Barcelona – Spain; 3 Spain Hospital Universitario de Badajoz, Badajoz – Spain; 4 Spain Hospital Universitario de la Santa Creu i Sant Pau, Barcelona – Spain; 5 Spain Hospital Universitario Clínico San Carlos, Madrid – Spain

**Keywords:** Arteries Dissection, Acute Coronary Syndrome, Gender Diversity, Fibromuscular Dysplasia, Multicenter Study

## Introduction

Spontaneous coronary artery dissection (SCAD) is an infrequent but increasingly recognized cause of acute coronary syndrome (ACS) that mainly affects women. It is the main cause of ACS during pregnancy and in up to one-third of women > 50 years old.^1 , 2^Owing to a unique demographic profile and a low prevalence of traditional cardiovascular risk factors, its cause appears to be multifactorial, with contributions from genetic factors, hormonal influences, inherited or acquired arteriopathies, and systemic inflammatory diseases.^1^SCAD is defined as a spontaneous – non-iatrogenic – separation of the coronary artery wall layers. Two main mechanisms could explain the pathophysiology of SCAD. In the first one, the primary event is hemorrhage within the media without intimal rupture; in the second mechanism, the primary event is the formation of a tear leading to an intimal flap. This initial disruption leads to the formation of an intramural hematoma or a true and false lumen that may cause myocardial ischemia.^1 , 2^Our understanding of this disease has increased greatly in the last decade as a result of international research efforts.^1 , 2^However, since ~90% of the cases affect women, the characteristics of SCAD in men and the potential clinical differences between sex remain poorly established.^3^According to previous data, men with SCAD have different predisposing and precipitating factors compared to women. The presence of fibromuscular dysplasia (FMD) and an association with mental disorders seem to be less frequent in men, while intense physical exercise is most commonly observed as a trigger of SCAD in this group.^4 , 5^The aim of this study was to compare baseline characteristics, clinical presentation, angiographic features, management strategies, and hospital course between men and women with SCAD.

## Methods

The Spanish SCAD Registry (SR-SCAD) (NCT03607981) is a prospective nation-wide multicenter study on SCAD performed under the auspices of the Association of Interventional Cardiology of the Spanish Society of Cardiology. The specific protocol, as well as the case report form and informed consent, were approved by the Ethics Committee of the coordinating center (Hospital Universitario La Princesa, Madrid), according to current Spanish legislation. From June 2015 to April 2019, 344 consecutive patients with SCAD (387 lesions) were included from 31 Spanish centers. All coronary angiograms were carefully and jointly reviewed by two expert operators in a central core laboratory at the coordinating center using a predefined methodology for SCAD analysis.^6^After careful review of the angiograms and clinical data, 26 patients were excluded due to more likely alternative diagnoses. SCAD types were characterized using the Saw angiographic classification.^7^Lesions with a double-lumen appearance were classified as type 1. Type 2 lesions were defined as abrupt changes in arterial caliber with clear demarcation from normal diameter to diffuse narrowing. Lesions with focal narrowing, resembling atherosclerotic lesions, were classified as type 3. For the definition of other angiographic patterns such as the “stick insect” or “radish” morphologies, the initial description by Motreff et al.^8^was followed. The presence of other angiographic findings suggestive of SCAD, such as a “broken line” pattern, was also systematically evaluated. Finally, the analysis of coronary tortuosity was performed according to the Mayo Clinic definition.^9^

Continuous variables are expressed as means ± standard deviations, and categorical variables are expressed as frequencies and percentages. For comparison, chi-square or Fisher’s exact tests were used as required for categorical data, and the Student’s t-test was used for continuous variables.

## Results

A total of 39 men and 279 women with SCAD ( Table 1 ) were compared. Age was similar in both groups and most patients had some classic cardiovascular risk factors. Recreational drug consumption was significantly higher in men (26% vs 3%; p < 0.01), while hypothyroidism was more common in women (15% vs 3%; p = 0.04). Emotional and physical stress triggers were numerically more frequent among women and men, respectively, but without statistically significant differences. Most patients presented with non-ST elevation myocardial infarction. Men more frequently had ventricular arrhythmias as the onset symptom (8% vs 1.4%; p = 0.01) and during hospitalization (5% vs 0.4%; p < 0.01) when compared to women ( Table 1 ). There were no differences in the detection of extracoronary vascular anomalies (EVA) or FMD between the men and women who underwent screening for this pathology.

**Table 1:** Sex differences in SCAD: baseline characteristics and clinical presentation

	Men (n=39)	Women (n=279)	p
Age (years)	50±10	54±11	0.3
Some CV risk factor	32 (82%)	216 (77%)	0.5
*Hypertension*	12 (31%)	106 (38%)	
*Dyslipidemia*	14 (36%)	97 (35%)	
*Diabetes mellitus*	2 (5%)	14 (5%)	
Recreational drugs	10 (26%)	9 (3%)	<0.01
Hypothyroidism	1 (3%)	41 (15%)	<0.01
Depression	8 (20%)	57 (20%)	1
Anxiety	4 (10%)	51 (18%)	0.2
Emotional stress	5 (13%)	74 (26%)	0.06
Intense physical exercise	7 (18%)	36 (13%)	0.4
NSTEMI	20 (51%)	150 (54%)	0.8
STEMI	12 (31%)	113 (40%)	0.3
Ventricular arrhythmias			
*Onset symptom*	3 (8%)	4 (1,4%)	0.01
*Hospitalization*	2 (5%)	1 (0,4%)	<0.01

*CV: cardiovascular; NSTEMI: non-ST segment elevation myocardial infarction; STEMI: ST segment elevation myocardial infarction.*

Notably, angiographic characteristics were significantly different between sexes. Men were more likely to have mild angiographic coronary irregularities compatible with associated coronary atherosclerosis in other vascular territories (15% vs 4%; p < 0.01), while they less frequently presented coronary tortuosity (36% vs 72%, p < 0.01). Furthermore, some characteristic angiographic patterns previously described in this entity^8^(such as the “inverted radish” morphology and termination of the SCAD just before the origin of a side branch) were more frequently observed in men. Conversely, the “stick insect” and “broken line” morphologies mainly occurred in women. ( Table 2 ) ( Figure 1 ).

**Table 2:** Sex differences in SCAD: angiographic features

	Men (n=39)	Women (n=279)	p
IVUS/OCT	8 (20%)	25 (9%)	0.03
LAD	16 (41%)	133 (48%)	0.9
Coronary atherosclerosis	6 (15%)	10 (3,6%)	<0.01
Coronary tortuosity	14 (36%)	201 (72%)	<0.01
Saw type 1	11 (28%)	59 (21%)	0.4
Saw type 2	22 (56%)	172 (62%)	0.5
“Radish” morphology	3 (7,7%)	41 (15%)	0.2
“Inverted radish” morphology	6 (15%)	12 (4%)	<0.01
SCAD ends before the origin of lateral branch	4 (10%)	7 (2,5%)	0.03
“Broken line” morphology	1 (3%)	52 (19%)	0.01
“Stick insect” morphology	0 (0%)	26 (9%)	0.05

*IVUS: intravascular ultrasound; LAD: left anterior descending artery; OCT: optical coherence tomography; SCAD: Spontaneous coronary artery dissection.*

**Figure 1:** Upper panel. (A-C) Right (A) and left (C) cranial angiographic views in a 45-year-old man, with no cardiovascular risk factors, admitted for NSTEMI. A long lesion is seen in the mid-LAD, characteristic of a spontaneous coronary artery dissection type 2a IMH (delimited by dashed white lines) that also affects the ostium of the second diagonal branch (black asterisk). The IMH begins and ends after the origin of two septal branches (S). In the final portion of the hematoma, there is evidence of an abrupt change in vessel caliber due to the compression generated by the IMH itself and distal recovery of the normal caliber. This morphology is reminiscent of a radish root (“radish” morphology) (B) and is described in this entity. The absence of significant coronary tortuosity in this man with SCAD is also verified. Lower panel. (D-F) Angiographic projections with cranial angulation of a 65-year-old woman, ex-smoker, hypertensive and dyslipidemic, admitted for NSTEMI with electrocardiographic data suggestive of anterior ischemia. (D) Caliber reduction in the mid-LAD and a long lesion in the first diagonal branch (limited by white arrows) compatible with a type 2a IMH. We highlight the presence of a healthy distal segment of the LAD with significant tortuosity, while the affected diagonal branch presents a marked “rectification” of smooth angles in the arterial curvature, with a “broken line” pattern (broken yellow line), which is also described in this pathology. The direct implantation of a drug-eluting stent (E, asterisks) was the treatment of choice, with a good final result (F, segment delimited by yellow lines) but with discrete worsening in the diagonal branch due to extension of the IMH. IMH: intramural hematoma; LAD: left anterior descending coronary artery; NSTEMI: non-ST-segment elevation acute coronary syndrome; SCAD: Spontaneous coronary artery dissection.

Treatment was primarily conducted through conservative medical management in both groups. However, percutaneous coronary intervention (PCI) was used as primary treatment in 22% of women and 23% of men. The most common PCI procedures were drug-eluting stent implantation (61% and 67% in women and men, respectively), plain balloon angioplasty (20% in women and 22% in men), and bioresorbable device implantation (13% in women and 11% in men) ( Table 3 ). The success rate of PCI was high in both sexes (86% and 100% in women and men, respectively), without differences between them. No sex differences were observed in the incidence of the combined clinical endpoint (which included hospital death, reinfarction, heart failure, and stroke) when considering both the conservative and interventional management options ( Table 3 ). Regardless of the management strategy, treatment at discharge was also similar for both sexes, although beta-blockers were more commonly used in women (81% vs 65%; p = 0.03).

**Table 3:** Sex differences in SCAD: management strategies and hospital course

	Men (n=39)	Women (n=279)	p
Presence of EVA (FMD included)	2/8 (25%)	29/85 (34%)	0,2
Conservative treatment	30 (77%)	217 (78%)	0,9
Interventional treatment	9 (23%)	61 (22%)	0,8
*Drug-eluting stent*	6 (67%)	37 (61%)	
*Balloon angioplasty*	2 (22%)	12 (20%)	
*Bioresorbable device*	1 (11%)	8 (13%)	
Adverse hospital event *	4 (10%)	14 (5%)	0,2

EVA: extracoronary vascular anomalies; FMD: fibromuscular dysplasia. * Includes: death, reinfarction, heart failure, and stroke.

## Discussion

Considering the scarcity of information on SCAD in men, this prospective nationwide registry provides interesting novel data that complements previous evidence. The most important findings of this study are the differences between men and women regarding precipitating factors and angiographic findings. Consumption of recreational drugs and ventricular arrhythmias were more frequently found in men. The higher prevalence of hypothyroidism among female participants is probably just a reflection of the fact that hypothyroidism predominantly affects women. Regarding angiographic findings, men often showed irregularities (suggesting the possibility of associated underlying mild atherosclerosis) and significantly less coronary tortuosity than women. There were also differences in certain angiographic patterns that are typical of this entity. The “inverted radish” morphology and the termination of the SCAD just before the origin of a side branch were more frequent in men, while the “stick insect” and “broken line” morphologies occurred mainly in women. Although the percentage of patients screened for EVA in our registry was relatively low (29% of all patients), no differences were found in the incidence of EVA between men and women with SCAD. Importantly, the lack of a systematic screening study in this regard with men could be due to the perception that FMD mostly affects women. However, Fahmy et al.^4^suggested the importance of systematic EVA screening in men, similar to what is currently recommended for women.^2^In line with contemporary literature, adverse events were infrequent in both groups and patients had good in-hospital outcomes, most of them under conservative management. Nevertheless, PCI is sometimes necessary and seems to be a good option for selected high-risk patients, with no differences in hospital course between men and women. However, larger studies and longer follow-up periods are still needed to assess potential differences in outcomes between sexes.

Prior studies comparing men and women with SCAD were single-center retrospective studies involving a smaller number of patients.^4 , 5 , 10^This work is the first prospective, nationwide, multicenter SCAD study including a larger number of patients (39 men and 279 women) and focused on sex differences. In some sex comparative studies,^4 , 5 , 10^men with SCAD were younger than women. However, no age-related differences were found in our cohort. As SCAD is not an atherosclerotic disease, it seems reasonable to expect a similar age range for men and women. It is still possible that a SCAD diagnosis is more frequently overlooked in older men. As in prior reports,^4 , 5^drug abuse was identified as a trigger for SCAD in men. However, in contrast to prior studies, no significant differences in physical and emotional stressors were seen in our study.^4 , 5^

Sharma et al.^5^suggested that men were more likely to present with a double-lumen angiographic appearance (71% type 1 SCAD vs 21% type 2 SCAD). Nevertheless, in our study as in prior reports,^1 , 2^type 2 SCAD was the most prevalent angiographic pattern in both sexes. It is reasonable to speculate that men with SCAD patterns that are different from the classical angiographic “dissection” are more frequently underdiagnosed. Motreff et al.^8^described specific angiographic patterns in women with SCAD. For the first time, we described these angiographic patterns in relation to sex. Even though the underlying mechanisms by which these unique angiographic morphologies are generated remain unknown, the identification of these interesting patterns may help in the differential diagnosis and might also suggest potential pathophysiological differences between men and women with SCAD.

Some limitations of this observational study should be acknowledged. First, although this is a large (the largest to date in Europe) prospective and multicenter study, SCAD is a rare condition, and some selection biases may have occurred due to overlooked diagnosis and a relatively small sample size. Second, the percentage of patients screened for EVA in our registry was low. Third, intracoronary imaging was not routinely obtained in this registry despite its diagnostic value and potential to help understand angiographic sex differences. Fourth, SCAD is probably underdiagnosed in older men and when its presentation has a non-classic angiographic pattern. Finally, only events that took place during hospitalization were analyzed. Although this nationwide registry aims to obtain long-term clinical follow-up data, this information is not currently available.

The identification of differences between men and women with SCAD provides novel insights in our understanding of the pathology of this unique clinical entity and ACS in general. We should keep in mind that SCAD also affects men and both sexes benefit from initially conservative management, with an excellent hospital outcome.
